# In Situ Identification of Secondary Structures in Unpurified *Bombyx mori* Silk Fibrils Using Polarized Two-Dimensional Infrared Spectroscopy

**DOI:** 10.1021/acs.biomac.2c01156

**Published:** 2022-11-28

**Authors:** Giulia Giubertoni, Federico Caporaletti, Steven J. Roeters, Adam S. Chatterley, Tobias Weidner, Peter Laity, Chris Holland, Sander Woutersen

**Affiliations:** † Van’t Hoff Institute for Molecular Sciences, University of Amsterdam, Science Park 904, 1098 XHAmsterdam, The Netherlands; ‡ Van der Waals-Zeeman Institute, Institute of Physics, University of Amsterdam, 1098 XHAmsterdam, The Netherlands; § Department of Chemistry, Aarhus University, 8000Aarhus C, Denmark; ∥ Department of Materials Science and Engineering, University of Sheffield, Sir Robert Hadfield Building, Mappin Street, SheffieldS1 3JD, U.K.

## Abstract

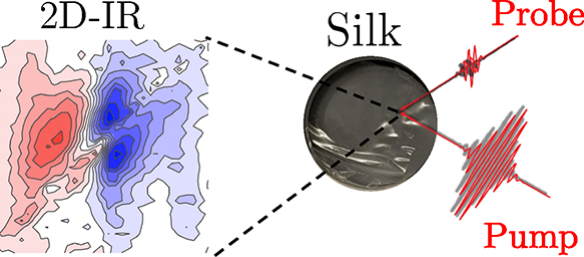

The mechanical properties of biomaterials are dictated by the interactions and conformations of their building blocks, typically proteins. Although the macroscopic behavior of biomaterials is widely studied, our understanding of the underlying molecular properties is generally limited. Among the noninvasive and label-free methods to investigate molecular structures, infrared spectroscopy is one of the most commonly used tools because the absorption bands of amide groups strongly depend on protein secondary structure. However, spectral congestion usually complicates the analysis of the amide spectrum. Here, we apply polarized two-dimensional (2D) infrared spectroscopy (IR) to directly identify the protein secondary structures in native silk films cast from *Bombyx mori* silk feedstock. Without any additional peak fitting, we find that the initial effect of hydration is an increase of the random coil content at the expense of the helical content, while the β-sheet content is unchanged and only increases at a later stage. This paper demonstrates that 2D-IR can be a valuable tool for characterizing biomaterials.

## Introduction

**Figure 1:**
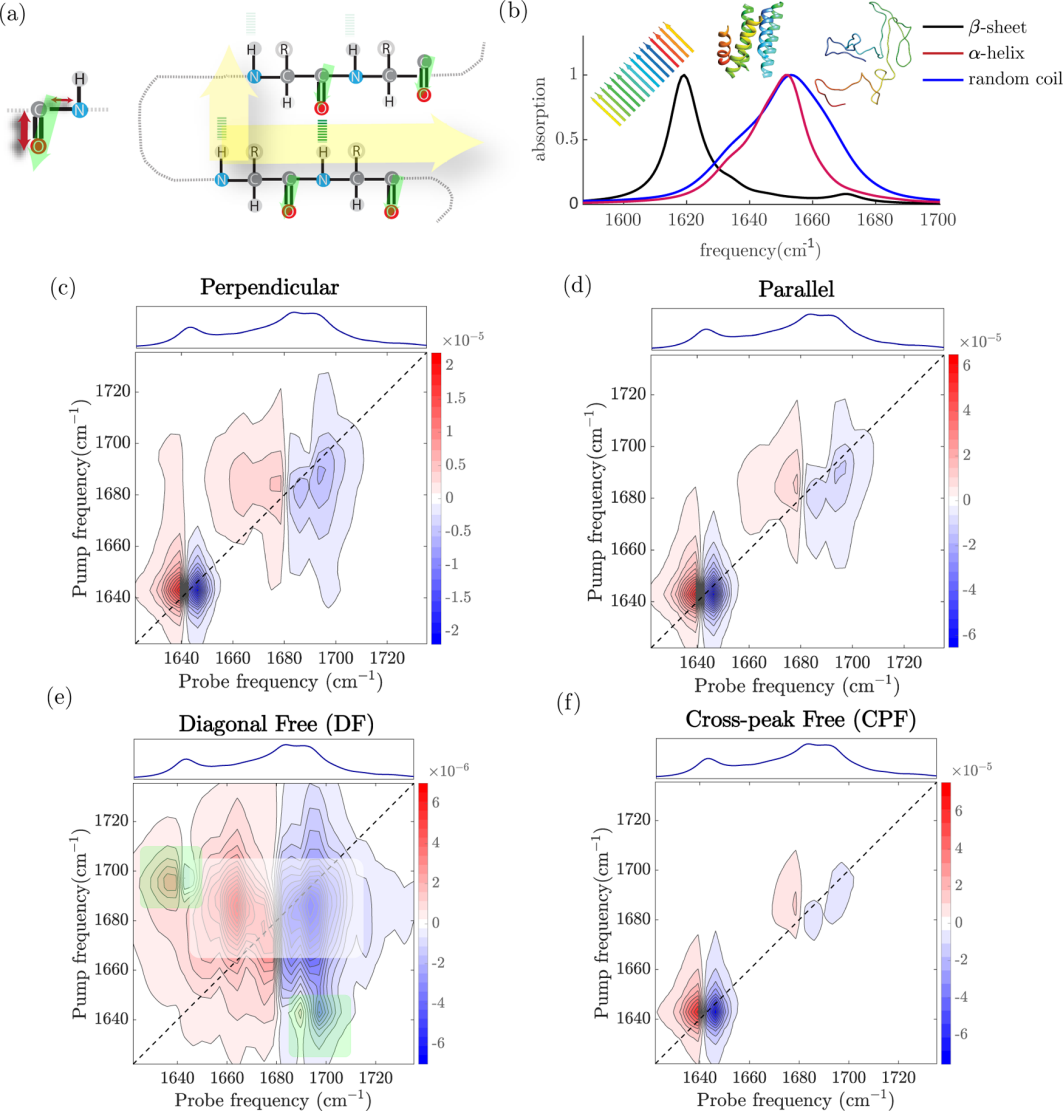
(a) Schematic of amide I vibrational modes in a single molecule and in a series of coupled amide groups. (b) Simulated linear spectra of short peptides. (c–f) Simulated 2D-IR spectra for a short peptide adopting β-sheet, random coil, and α-helix conformations. Color coding: Δ*A* < 0 blue and Δ*A* > 0 red. (e) Diagonal-free 2D-IR spectrum is obtained by subtracting the (3×) scaled perpendicular (c) to parallel (d), while cross-peak free spectrum (f) is obtained by subtracting parallel (d) to two times perpendicular (c). The green and white rectangles in (e) show the position of the cross-peaks associated with the β-sheet and α-helix secondary structures, respectively.

The mechanical properties of multiscale hierarchical biomaterials, such as the rigidity of bones or the toughness of spider silk, are dictated by the molecular properties of their building blocks that self-assemble to form ordered hierarchical supramolecular structures.^1,2^ Among the different proteins that act as biomolecular building blocks, silk proteins (i.e., fibroin from silkworms or spidroins from spiders) are among the most extensively studied and have become a model biopolymer system, as a result of their accessibility for research and outstanding mechanical and biocompatible properties.^3^ Silk proteins self-associate to form heteronanocomposites and highly hierarchical supramolecular fibrils. These fibrils are composed of ordered nanocrystals embedded in disordered amorphous regions.^4^ The molecular properties of the fibrils, such as the adopted secondary structure and the crystal size,^5^ dictate the mechanical properties of silk. The β-sheet appears to be the most stable form and dominates the crystalline content of natural silk fibers,^6^ while helical and random structures can be observed in the noncrystalline material.^7^ Less stable helical and random coil structures dominate films cast from water by evaporation under mild conditions, assumed to reflect the prevalence of random coil conformations in aqueous solution.^8−12^ Upon exposure to high humidity, the increased water content causes glass-transition-induced softening and an increase in extensibility, as helical structures can be converted to swollen random coil structures, while the β-sheet content can increase.^7^ Multiple techniques have been applied to study the silk–protein structure under different humidity conditions, providing a clear understanding of the adopted conformation in the nanocrystals. However, the molecular arrangement in the amorphous regions is still debated, principally due to the fact that for most structure-sensitive techniques, it is challenging to identify those regions, or the samples require harsh treatments that can strongly affect and change the protein structure. This problem can be avoided by infrared spectroscopy, which is a label-free and noninvasive technique that is widely used to investigate the structure and conformation of biomolecular building blocks.^13^ The molecular vibrations of the amide groups, in particular the amide I mode that involves the carbonyl stretching (Figure 1a), are sensitive to the protein conformation. In β-sheet and α-helix structures, amide groups are connected by hydrogen bonds, leading to a long-range order along the protein backbone and couplings between the amide vibrations, which are mostly of a dipolar nature.^13−17^ These couplings give rise to delocalized normal modes, and for both ideal β sheets and α helices, the two most important IR-active normal modes have perpendicular transition dipole moments.^13,18^ In the case of antiparallel β-sheets,^13,16,19^ the so-called A_⊥_ and A_∥_ modes absorb at 1620–1630 and 1680–1700 cm^–1^, respectively.^13,16,20^ In the case of an α-helix, the frequency difference between the parallel (A) and perpendicular (E) modes is typically only few wavenumbers,^18,21^ and the A and E bands have significant overlap (Figure 1b), resulting in a single band centered at ∼1650–1660 cm^–1^.^13^

Unfortunately, the infrared absorption spectra in the amide I region are generally rather congested, limiting our ability to disentangle the underlying amide I bands in an unambiguous manner. Many indirect methods of data analysis have been developed to solve this problem.^22^ However, identifying secondary structures based only on the absorption frequencies obtained via fitting may not be sufficient to determine the secondary structures present. Indeed, an assignment based on the absorption frequency is not always unique because of secondary effects, such as solvent interactions, that may shift the amide I vibrational frequencies, and more specific spectral signatures are required. To overcome these problems, we use two-dimensional infrared spectroscopy (2D-IR) to investigate the secondary structure of silk. 2D-IR spectroscopy can detect couplings between vibrational modes directly and use these to obtain structural information^23−29^ in a manner somewhat similar to 2D-NMR, where spin–spin couplings are detected and used to obtain structural information.^30^

Here, we apply 2D-IR spectroscopy to investigate in situ the secondary structures present in films produced using unpurified fibroin from *Bombyx mori* silkworms. By selecting specific polarization combinations, we obtain unique spectral signatures that allow us to disentangle and assign vibrational bands to specific secondary structures. We find that the film contains helical, β-sheet, and random coil structures, with the helical structure being most predominant at ambient humidity. The exposure of the film to a saturated water environment leads to a decrease in the helical contribution and an increase in the spectral region of the β-sheet and random coil conformations. This work demonstrates that 2D-IR spectroscopy can be used, without peak fitting, to measure structure conversion in a hydrated silk film. Thus, we highlight the unique ability of 2D-IR to characterize the protein secondary structure in biomaterials in a direct manner, opening a new set of potential interdisciplinary applications for 2D-IR.

## Methods

### Silk Film Preparation

Films were prepared using the native silk feedstock (NSF) from the middle–posterior (MP) sections of silk glands from commercially reared *B. mori* silkworms (four-way polyhybrid cross of two Japanese and two Chinese strains) in their 5th instar. Specifically, silkworms during the early stages of cocoon construction were sacrificed by decapitation, allowing the two silk glands and hemolymph to be ejected into a Petri dish.

One gland was selected and transferred to a second Petri dish and immersed in type I (ultrapure distilled and deionized water, with resistivity around 18.2 MΩ·cm) water. Using a pair of tweezers, the gland was divided around the midpoint, and the anterior portion (containing more sericin) was discarded. A second cut was made where the (wider) middle section started, and the (relatively narrow) posterior section was also discarded. The thin membrane was peeled off the MP gland section, using fine tweezers under a stereomicroscope, and the viscous NSF (around 0.15 g, containing around 0.035 g of predominantly fibroin containing less than 3% w/w sericin) was transferred to a polystyrene weighing boat. Around 2 to 3 mL of type I water was added, and the weighing boat was loosely covered with tissue paper and allowed to stand at ambient temperature. The NSF was initially dissolved into the water, and then a film formed as the water evaporated. The film was allowed to dry under ambient conditions for a few days, before being transferred to a vacuum oven (still in the weighing boat). Drying to constant weight was completed over several hours at 60 °C under vacuum. Then, the film was peeled off the weighing boat and transferred to a sealed plastic bag for storage until required.

### FTIR and 2D-IR Spectroscopy

Samples for infrared spectroscopy were prepared by selecting a ∼1 cm^2^ part of a ∼10 μm thick silk film and placing it between two CaF_2_ windows in a customized cylindrical sample holder. After the measurements at ambient humidity (<60%), the sample cell was partially disassembled by removing the top window, and it was placed for 120 min in a D_2_O environment at a relative humidity of ∼85%. The humidity inside the hydration chamber was controlled, over time, using a digital hygrometer. The silk was hydrated using D_2_O to avoid heating effects, in the ultrafast experiments, due to the bending mode of H_2_O, which overlapped with the amide band.^31^

A Perkin-Elmer Spectrum-Two FTIR spectrometer (resolution 4 cm^–1^) was used to measure the FTIR spectra. A detailed description of the setup used to measure the 2D-IR spectra can be found in ref (32). Briefly, pulses of a wavelength of 800 nm and with a 40 fs duration were generated by a Ti:sapphire oscillator and further amplified by a Ti:sapphire regenerative amplifier to obtain 800 nm pulses at a 1 kHz repetition rate. These pulses were then converted in an optical parametric amplifier to obtain mid-IR pulses (∼20 μJ, ∼6100 nm) that had a spectral full width at half-maximum (FWHM) of 150 cm^–1^. The beam was then split into probe and reference beams (each 5%) and a pump beam (90%) that was aligned by a Fabry–Pérot interferometer. The pump and probe beams overlapped in the sample in an ∼250 μm focus. The transmitted spectra of the probe (*T*) and reference (*T*_0_) beams with the pump on and off were then recorded after dispersion by an Oriel MS260i spectrograph (Newport, Irvine, CA) onto a 32-pixel mercury cadmium telluride (MCT) array. The probe spectrum was normalized to the reference spectrum to compensate for pulse-to-pulse energy fluctuations. The 2D-IR signal was obtained by subtracting the probe absorption in the presence and absence of the pump pulse. Parallel and perpendicular 2D-IR spectra were recorded by rotating the pump beam at a 45° angle with respect to the probe beam and selecting the probe beam component that was either perpendicular or parallel to the pump beam using a polarizer after the sample. To minimize pump-scattering contributions, we measured the average of two photoelastic modulator (PEM)-induced pump delays, such that the interference between the scattered pump beam and the probe beam had a 180° phase in one delay with respect to the other delay.

### Spectral Calculations

The spectral calculations were performed in accordance with the formalism described in ref (25, 33). We used the transition dipole coupling (TDC) model^34^ to determine the couplings between the amide I modes in the protein backbones. In this model, the through-space coupling was approximated by a Debye-like coupling that was dependent on the relative orientation and distance between the amide I oscillators. The orientation and magnitude of the isolated amide I transition dipole moments were determined by density functional theory (DFT) calculations using the BLYP functional in combination with the 6-311+G(2df,p) basis set and the IEFPCM water solvent mode on deuterated *N*-methyl acetamide (NMA).^35^

First, we applied this formalism to three different structures to calculate “pure component” 1D- and 2D-IR spectra for three types of secondary structure: random coils obtained from molecular dynamics simulations of α-synuclein,^36^ from X-ray crystallography experiments on the mainly α-helical structure of spindroid proteins, a component from spider silk, (PDB: 3LR2(37)) and from a perfect antiparallel β-sheet structure generated in Chimera^38^ using standard parameters for the dihedral backbone angles (ϕ = −139°, ψ = 135°). From the 73 μs α-synuclein simulation, we took 73 snapshots (frames), starting from frame 1, spaced by a simulation time of 1 μs (see Figure 1b for the first frame). We then calculated the 2D-IR spectra for each of the frames and determined the average 2D-IR spectrum of the ensemble to optimally sample the many conformations that a randomly coiled structure adopted. The α-helical 2D-IR spectra were calculated by taking just the α-helical structure of chain A of PDB 3LR2 and removing the three prolines from the sequence, together with the 2–3 residues after the proline residue at the ends of the three proline-containing helices. This latter step was performed to keep the “pure component” calculations as clean as possible; the amide I local mode of proline was red-shifted by ∼19 wavenumbers,^33^ which led to more complex amide I normal modes. Finally, the perfect antiparallel β-sheet was created by placing 16 polyalanine 16-mers in an antiparallel sheet, shifting each of them by 5 Å with respect to each other in the hydrogen-bonding direction of the β-sheet, by rotating every second β-strand monomer by 180° and shifting it by 1.5 Å to align the amide groups of the β-strands. Subsequently, to obtain the spectra depicted in Figure 1, we mixed these three pure component spectra in accordance with the fitted ratio of the treated experimental 2D-IR spectrum (i.e., a 0.6:0.21:0.19 α-helix/β-sheet/random coil ratio).

## Results and Discussion

### FTIR Spectrum

**Figure 2:**
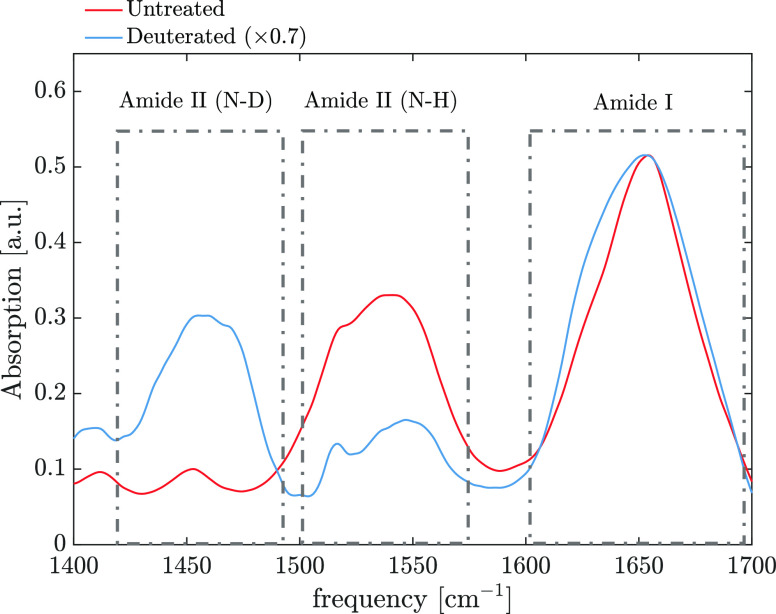
Infrared absorption spectra of a ∼10 μm thick silk film in the amide I and amide II absorption regions. The untreated sample was measured without further purification at ambient humidity (∼60 %). The untreated sample was then incubated at an RH of 85 ± 10% in a saturated D_2_O environment for 120 min.

In Figure 2, we show the infrared absorption spectra of untreated and hydrated silk films in the region between 1400 and 1600 cm^–1^. In this region, the most predominant absorption bands are two vibrational bands of the amide groups, amide I and amide II.^13^ The amide I mode originates mainly from the carbonyl stretching vibration and absorbs between 1600 and 1700 cm^–1^, and amide II is the “out-of-phase” or asymmetric combination of C–N stretch and N–H bending and absorbs between 1450 and 1550 cm^–1^. The untreated silk film shows a strong absorption band at 1655 cm^–1^, with a shoulder at a lower frequency of around 1625 cm^–1^. The presence of these bands indicates that silk contains more than one secondary structure. While the band at 1625 cm^–1^ can be assigned to β-sheet structures,^13^ the assignment of the main band at 1655 cm^–1^ is not straightforward: both random coil and helical structures can absorb at this frequency.^13^ The amide II vibrational mode is found at a lower frequency of 1550 cm^–1^. In the region between 1400 and 1500 cm^–1^, we also find different vibrational bands that we can assign to side-chain modes, such as CH bending. We later incubated the silk film in a saturated D_2_O environment for a period of 120 min at an RH of 85 ± 10%. The full measured infrared spectrum is also reported in Figure S1. With increasing D_2_O exposure, the shoulder at 1625 cm^–1^ increases in intensity, and most of the amide II band at 1550 cm^–1^ red-shifts to around 1480 cm^–1^. We calculate the areas of the untreated and deuterated amide II bands and find that ∼70% of the NH groups are H/D-exchanged to ND (see Figure S2), whereas it is clear that the exposure to a saturated D_2_O environment causes an increase in the shoulder at 1625 cm^–1^, suggesting, at first glance, an increase of the content of β-sheet structures. However, the congested infrared spectra do not directly provide information on the type of other secondary structures present in the film, and because of this, a quantitative estimation of the effect of increased humidity is not straightforward. To obtain a better understanding of the secondary structures in silk, we used two-dimensional infrared spectroscopy. Before discussing the 2D-IR results, we briefly explain the principle of this spectroscopic method.

### Principle of 2D-IR Spectroscopy

In a 2D-IR spectrum, vibrational couplings lead to specific spectral signatures (cross-peaks) that contain information on the coupling strength (which depends strongly on the distance between the coupled vibrating bonds) and on the angle between the transition dipole moments of the coupled modes.^23−25^ In pump–probe 2D-IR spectroscopy, an intense, narrow-band infrared pump pulse (with adjustable center frequency ν_pump_) resonantly excites at a specific frequency (in the present case, in the amide I band). A delayed, broad-band probe pulse is used to probe the frequency-dependent IR absorption change Δ*A*, which results from the excitation by the pump beam. Measuring the Δ*A* spectra for a range of ν_pump_ values, we obtain two-dimensional spectra showing the pump-induced absorption change Δ*A*(ν_probe_, ν_pump_) as a function of the pump and probe frequencies ν_pump_ and ν_probe_. The 2D-IR signal can be recorded with the pump- and probe–pulse polarizations parallel (∥) or perpendicular (⊥) to each other, and the polarization dependence of the cross-peak intensity is determined by the angle between the transition dipole moments of the coupled modes.^25^

As an example, we show in Figure 1c the simulated 2D-IR spectra in the amide I region of a short peptide adopting ideal β-sheet and α-helix structures. When the pump frequency is resonant with the ν = 0 → 1 frequency of either of the two β-sheet modes (at ∼1620 and 1670 cm^–1^), part of the molecules are excited to the ν = 1 state of this mode, resulting in a decrease in the absorption at the ν = 0 → 1 frequency (Δ*A* < 0 feature on the diagonal) and an increase in absorption at the ν = 1 → 2 frequency (which is at a slightly lower value than the ν = 0 → 1 frequency due to the anharmonicity of the vibrational potential), resulting in a Δ*A* > 0 feature slightly to the left of the diagonal. In this way, each normal mode gives rise to a ± doublet on the diagonal of the 2D spectrum (diagonal peaks).

If two normal modes A and B are coupled, then (to first-order approximation) exciting mode A causes a small red shift of the frequency of mode B,^25^ resulting in an absorption decrease on the high-frequency side of the B band and an absorption increase on the low-frequency side and so a ± doublet at (ν_probe_, ν_pump_) = (ν_B_, ν_A_), whose amplitude depends on the coupling strength. The intensities of the cross-peaks also depend on the angle between the pump and probe polarizations, in a way that is determined purely by the angle between transition dipole moments of the coupled modes. In particular, if the transition dipole moments of the coupled modes are perpendicular (as is the case for the IR-active modes of α-helices and β-sheets), then the relative intensity of the cross-peaks is higher in the perpendicular spectrum than in the parallel spectrum (Figure 1c). The set of diagonal and cross-peak features (and its polarization dependence) of α-helices and β-sheets forms a pattern in the 2D-IR spectrum that can be used as a fingerprint of these secondary structures.

#### Polarization-Dependent 2D-IR Spectra of *B. mori* Silk

**Figure 3:**
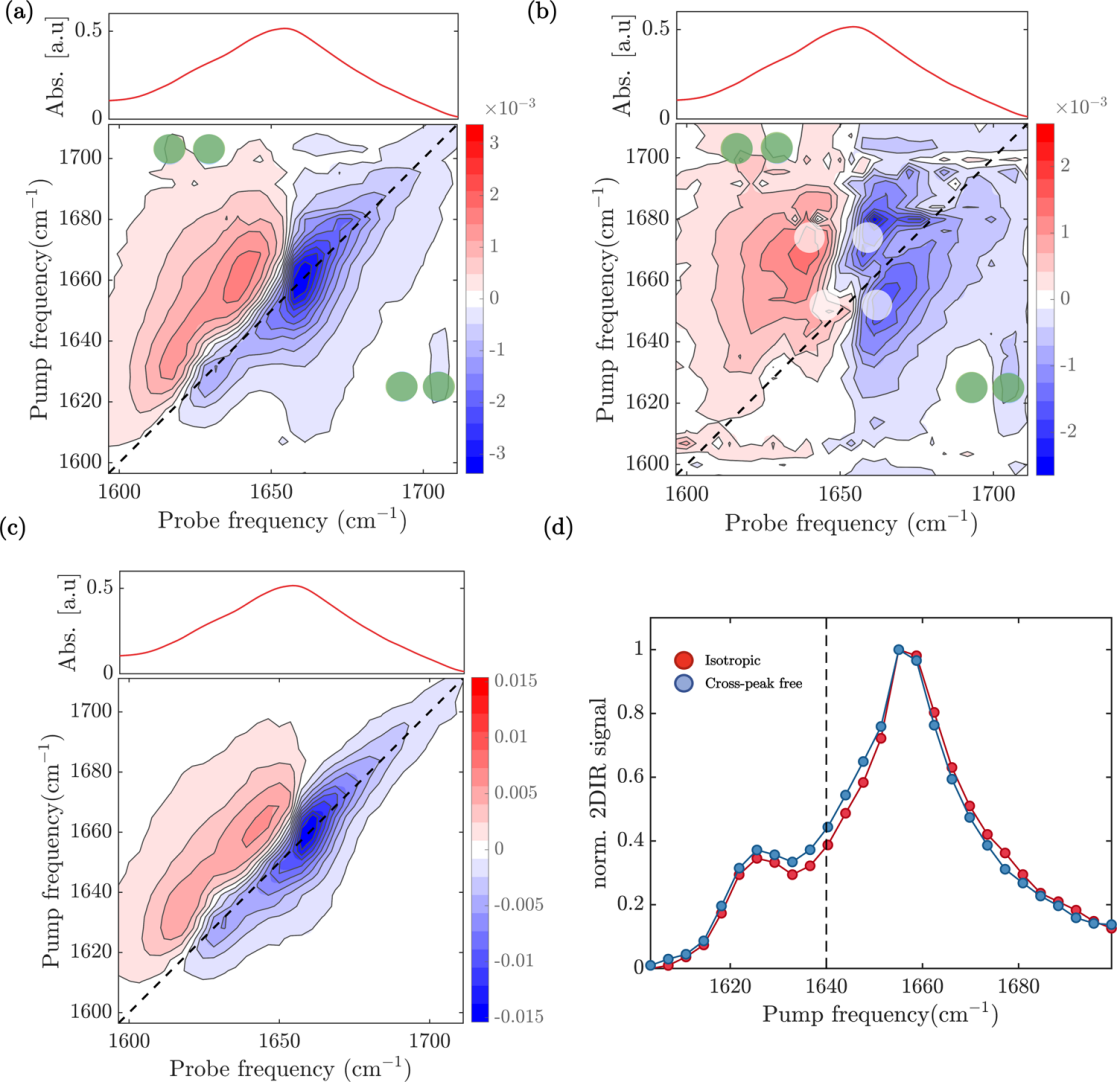
2D-IR analysis of the untreated silkworm film. (a) Perpendicular and (b) diagonal-free 2D-IR spectra at a time delay of 1 ps. Green and white dots indicate the positions of β-sheet and helical cross-peaks, respectively. (c) Cross-peak-free 2D-IR spectrum at a time delay of 1 ps. (d) Diagonal slices of the bleach signals of the isotropic (red circles) and cross-peak-free (blue circles) 2D-IR spectra at a time delay of 1 ps. The absorption appears to be enhanced across quite a wide range of wavenumbers, from around 1630 to 1650 cm^–1^.

In Figure 3a, we show the perpendicular 2D-IR spectrum of the untreated silk sample. We observe strong diagonal peaks when exciting at 1660 and 1630 cm^–1^. The diagonal peak at ν_pump_ = 1630 cm^–1^ corresponds to the weak shoulder observed in the FTIR spectrum. In the 2D-IR spectrum, this peak is better resolved because the 2D-IR signal scales with the square of the absorption cross section ∼σ^2^, whereas the FTIR signal scales as ∼σ.^25^ In the 2D-IR spectra, we can thus nicely resolve the presence of two well-separated vibrations along the pump frequency at 1660 and 1630 cm^–1^, which correspond to the vibrational bands observed in the FTIR spectra at 1655 and 1625 cm^–1^, respectively. The apparent slightly higher vibrational frequency in the 2D-IR spectra with respect to the FTIR is due to the experimental difference, such as a lower frequency resolution in the 2D-IR, for instance. The peaks colored in blue represent decreases in absorption (Δ*A* < 0) due to depletion of the amide I ν = 0 state, and the signal at a lower probe frequency colored in red represents the induced absorption of the ν = 1 → 2 transition. In the off-diagonal region, cross-peaks are visible. In particular, when exciting at 1630 cm^–1^, we observe a response at a probe frequency of 1700 cm^–1^. The negative half of the cross-peak ± doublet is not clearly visible due to overlap with the positive part of the (much stronger) diagonal peak. Typically, the cross-peaks in a 2D-IR spectrum are much less intense than the diagonal peaks, and often they partly overlap with them. To isolate the cross-peaks from the diagonal peaks we can use their different dependencies on the angle between the pump and probe polarizations. The diagonal peaks always have the same 3:1 intensity ratio for parallel versus perpendicular pump–probe polarizations, whereas for the cross-peaks, this intensity ratio is less than 3 (except in the special case where the transition dipole moments of the coupled modes are exactly parallel).^25^

Experimentally, a ratio different from 3 in the diagonal peaks can be found because of small polarization changes due to fluctuations in the pump and probe. However, the correct ratio can easily be retrieved by calculating the anisotropy from diagonal peaks not overlapping with cross-peak features, in our case, for instance, by the diagonal signal from the β-sheet mode.^39^ As a consequence, subtracting the perpendicular to parallel 2D-IR spectra (with an appropriate scaling factor *k*, which in the ideal case is 3), we obtain a diagonal-free (*A*_DF_) 2D-IR spectrum (see, as an example, the simulated spectrum in Figure 1e) and highlight only the cross-peaks.^40,41^
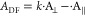
1Furthermore, the magnitude of these cross-peaks is proportional to the amount of protein adopting a certain specific secondary structure. Thus, we can use the relative intensity of the cross-peak to monitor the relative change in secondary structure content. Removing the diagonal peaks by carefully subtracting perpendicular to parallel is especially useful for observing α-helix cross-peaks because these strongly overlap with the diagonal peaks (as the frequency difference between the two coupled modes A and E is only a few wavenumbers.^42,43^) In a similar fashion, provided that the parallel/perpendicular intensity ratio of all cross-peaks in a 2D-IR spectrum is the same, we can suppress the cross-peaks by subtracting the appropriately scaled perpendicular 2D-IR spectrum from the parallel one,
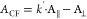
2obtaining a cross-peak-free 2D-IR spectrum where only the diagonal peaks are present, as exemplified by the simulation in Figure 1f.

**Figure 4:**
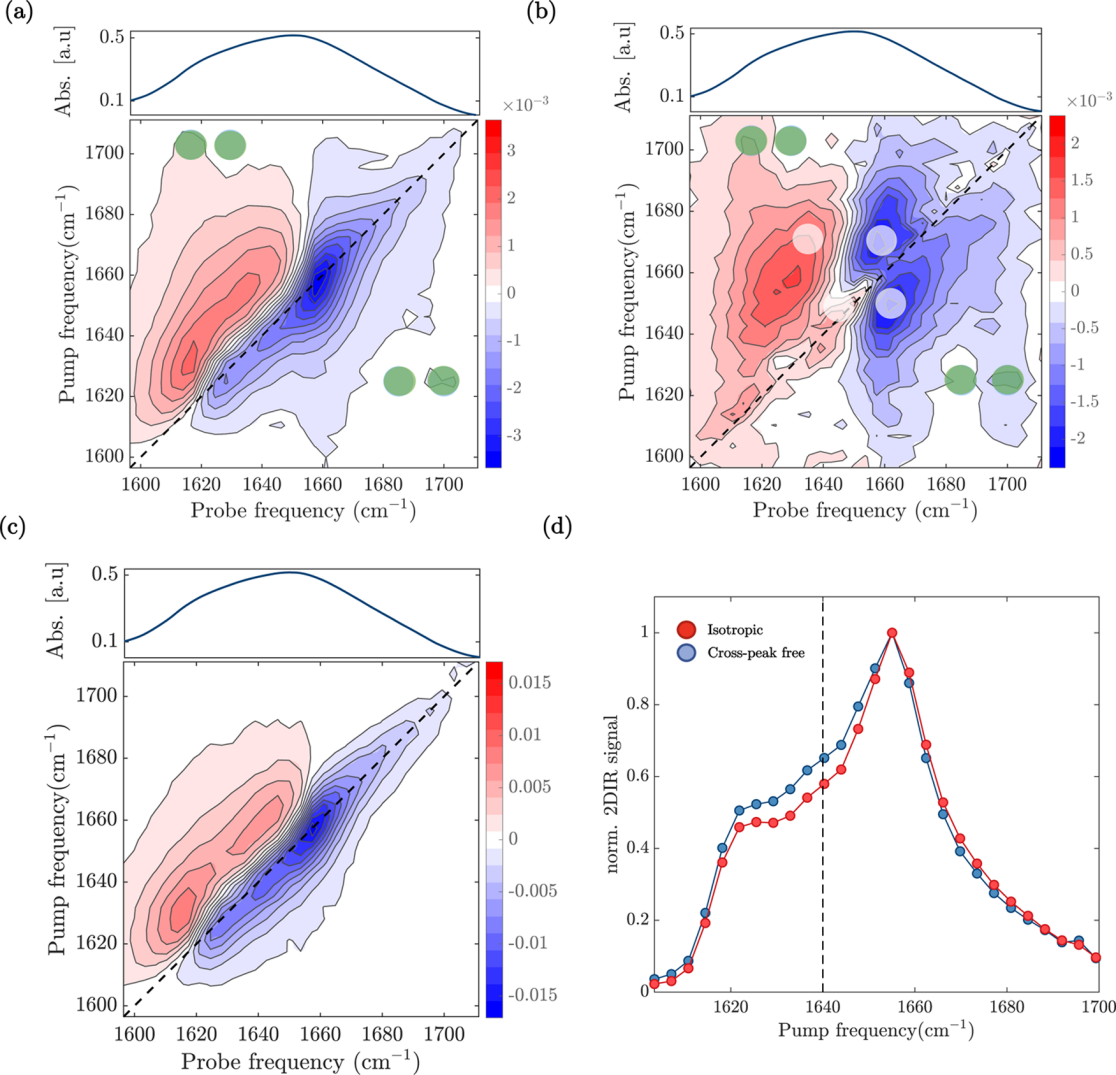
2D-IR analysis of the treated silkworm film. (a) Perpendicular and (b) diagonal-free 2D-IR spectra at a time delay of 1 ps. Green and white dots indicate the positions of β-sheet and helical cross-peaks, respectively. (c) Cross-peak-free 2D-IR spectrum at a time delay of 1 ps. The linear absorption spectra of the treated silkworm film are reported on top of the 2D-IR spectra (a–c) for comparison. (d) Diagonal slices of the bleach signals of the isotropic (red circles) and cross-peak-free (blue circles) 2D-IR spectra at a time delay of 1 ps. The vertical line shows the enhanced absorption from 1630 to 1650 cm^–1^.

Figure 3b shows the polarization-difference 2D-IR spectrum obtained by subtracting parallel from three times perpendicular (in our experiments, the parallel-to-perpendicular scaling factor is found to be 3 (Figure S3), in agreement with the theoretical value).^25^ We can now resolve the cross-peak between 1630 and 1700 cm^–1^ (indicated by green dots), which indicates the presence of β-sheet structures; 1630 cm^–1^ is the absorption band of the A_⊥_ mode, while 1700 cm^–1^ is of the A_∥_ mode. Interestingly, we also observe cross-peak signatures when excited at 1658 cm^–1^ and 1665 cm^–1^ (indicated by the white dots). As previously explained, the coupling of the amide I modes in an α-helix leads to two delocalized modes, defined as A and E. In the linear infrared spectrum and in the isotropic 2D-IR spectrum, these two modes cannot be resolved and give rise to a broad band centered around 1660 cm^–1^. However, since the A and E transition dipole moments are perpendicularly oriented to each other, the visibility of the cross-peaks between the A and E modes is enhanced when subtracting the parallel signal from three times the perpendicular signal. This procedure reveals the presence of two cross-peaks, with negative parts at ν_probe_ = 1658 cm^–1^ and 1665 cm^–1^. The frequency splitting of the A and E modes is around 10 cm^–1^. A recent study has suggested that part of silk proteins adopt a helical-like structure by forming repeated type II β-turns, which might also lead to a similar cross-peak pattern.^44^ Although we cannot completely exclude that a helical conformation originates from this proposed structure, the observed splitting is in agreement with values that were observed for α-helixes,^42^ supporting the assignment of the band at 1658 cm^–1^ to an α-helical structure. Figure 3c shows the 2D-IR spectrum obtained by subtracting the perpendicular signal from two times parallel signal (to be compared with the simulated one in Figure 1f). As discussed before, by doing so, we eliminate the cross-peaks between perpendicular modes. In Figure 3d, we show the diagonal slices of the bleach signals of the isotropic and cross-peak-free 2D-IR spectra. The cross-peak-free diagonal slice reveals the presence of the third band around 1640 cm^–1^ (see SI for reproducibility), which we assign to the random coil structure. Figure 4a,b shows the perpendicular and polarization-difference 2D-IR spectra of the hydrated silk film, respectively. We again observe the presence of two main diagonal peaks, obtained when excited at 1630 and 1660 cm^–1^. The peaks are much more elongated because of the increased inhomogeneous broadening, reflecting the structural disorder caused by the increased hydration of the amide groups.^25^ In the diagonal-free 2D-IR spectrum shown in Figure 4b, we notice the same cross-peak features observed previously, indicating that upon hydration, the silk film maintains α-helical and β-sheet conformations. We observe, however, that the increased hydration lowers the frequency of the highest vibrational band and broadens the cross-peak of the β-sheet structure: in the untreated sample, A_∥_ absorbs at 1705 cm^–1^, while in the hydrated sample at 1700 cm^–1^. The frequency shift and broadening indicate that part of the β-sheet structures become hydrated, likely in the interphase part between crystalline and amorphous regions,^20^ though the observed red shift of the band could be partially due to H/D exchange.^45^

To better resolve the diagonal signatures, we again subtract scaled parallel to perpendicular to remove the cross-peaks (Figure 4b). The diagonal slices of the cross-peak-free and isotropic signals again reveal the presence of three bands (1630, 1640, and 1660 cm^–1^).

**Figure 5:**
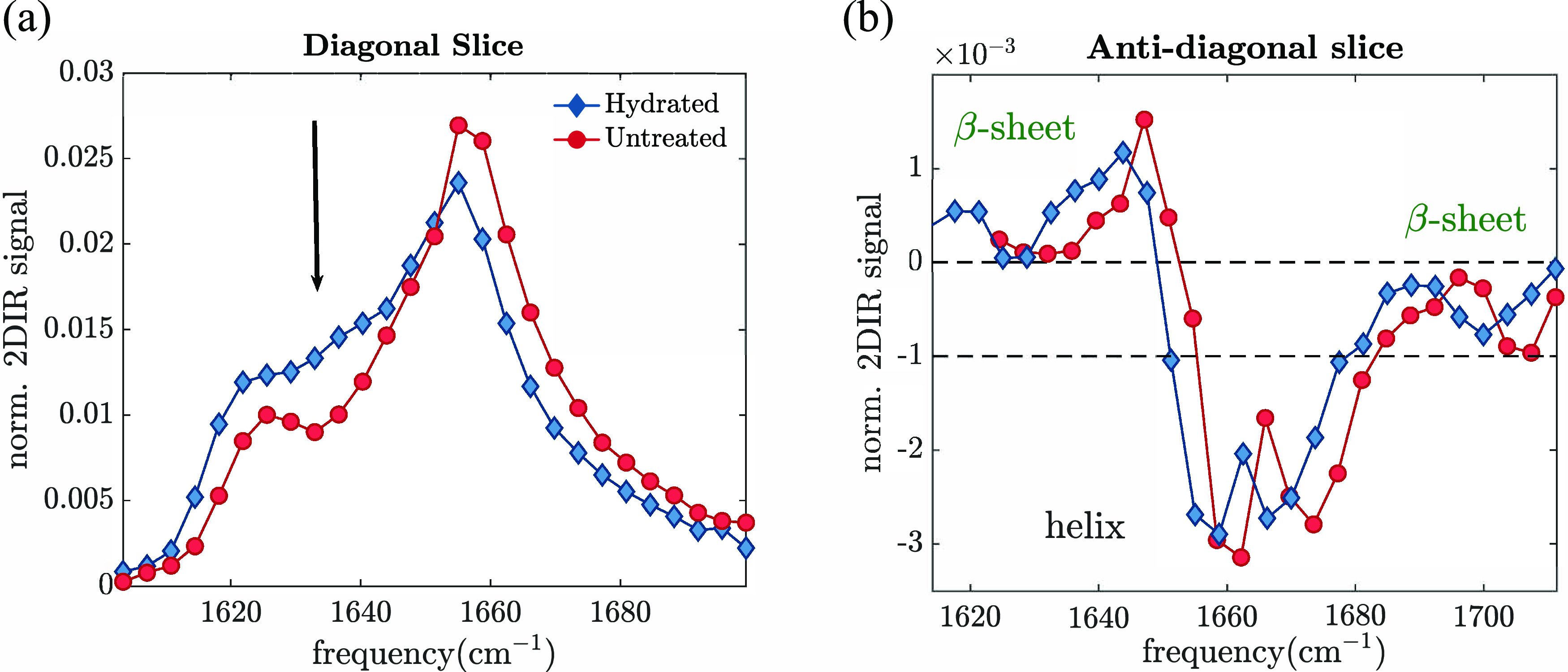
Untreated and treated isotropic diagonal (a) and antidiagonal slices (b). (a) Diagonal slices obtained from the 2D-IR spectra shown in Figures 3c and 4c, normalized in area. (b) Antidiagonal slices of the 2D-IR spectra shown in Figures 3b and 4b. The spectra have been normalized to the area of the diagonal-free spectrum.

In Figure 5a, we report the cross-peak-free diagonal slices of the treated and untreated samples, which we normalize to the respective spectrum areas. Compared to the linear infrared spectra, we can now better resolve the individual vibrational bands. This is because the 2D-IR signal scales as the cross section σ^2^, while the linear IR signal scales as σ, leading to narrower peaks.^46^ In the untreated spectrum, the 2D-IR signal shows a strong absorption band centered at 1660 cm^–1^, which we assigned before to the helical structure. At ambient humidity, we find that the dominant secondary structure is helical (this is reproduced in a second sample, see Figure S4). Upon increasing the humidity, the helical peak decreases in intensity, while the 2D-IR signal around 1640 and 1620 cm^–1^ increases. This observed increase in absorption might be partly due to the band shift caused by the change in the vibrational frequencies of helical and/or random coil due to H/D exchange.^45^ However, the shift of the helical amide I band due to H/D isotope exchange is usually around 1–2 cm^–1^^45^ and hence not sufficient to explain the observed increase in the diagonal bleach around 1640 cm^–1^. On the other hand, the random coil band, on average, can shift 8–9 cm^–1^, and thus, an increase in the signal at 1640 cm^–1^ might be partly due to a shift of an underlying band around 1650 cm^–1^, which we cannot resolve. Although we cannot exclude such an effect, the similar absorption at 1650 cm^–1^ (reproduced in the spectrum of Figure S11) in the two states suggests that the hydration causes a spectral change in which the absorption in the low-frequency region between 1620 and 1640 cm^–1^ increases at the expense of the 1660 cm^–1^ band. Such absorption enhancement is thus highly likely related to an increase in β-sheet and/or random coil contents. In this case, determining the relative contribution of these two structures to the observed increase is not easy. In fact, the two vibrational bands strongly overlap with each other, and there is no major change at one single absorption frequency, specific for either the random coil or β-sheet mode. Multipeak fitting analysis will, hence, lead to ambiguous results in this case. To solve this problem, we use the diagonal-free 2D-IR spectra, where the β-sheet and helical cross-peaks are well-resolved, while there is no contribution from the random coil. In fact, by studying the cross-peaks, we can directly estimate whether the number of β-sheet structures increases, since the magnitude of these cross-peaks scales with the β-sheet content. The intensity of the β-sheet cross-peak can be easily extracted from the antidiagonal slice of the 2D-IR spectrum, which passes through the bleach of the cross-peak. The peaks appearing in such an antidiagonal slice reflect the content of secondary structures, and hence, if the number of β-sheet structures increases, we can expect more pronounced (i.e., more negative) peaks in the antidiagonal spectrum of the hydrated film than those in the untreated one. Figure 5b shows the antidiagonal slices obtained from the diagonal-free 2D-IR spectra (Figure S5). We normalize them to the area of the respective cross-peak-free diagonal slices. We observe that both β-sheet cross-peaks absorbing around 1625 and 1700 cm^–1^ do not change significantly, suggesting that the β-sheet content stays constant, whereas the random coil increases. Further measurements performed on a new sample from the same batch and using a different experimental setup support this scenario (see Figures S6, S10, and S11 and Supporting Section “Measurement Reproducibility”). Longer exposure to humidity leads to a clear increase in the β-sheet structure (see Figure S6), in agreement with the literature.^47^ A previous study^47^ suggests that water weakens the hydrogen bonds within the helical structures, enabling chain movement and β-sheet formation because of helix–helix interactions, similar to what happens in amyloid formation. The fact that we observe an increase of random coil in a short exposure time to D_2_O might suggest the possibility that the helical-to-β-sheet structure conversion requires a critical hydration level, which is not reached in the first 2 h. However, hydration might be enough to lead to the unfolding of the helix into the random coil.^47^

As a comparison with the 2D-IR data, we calculated the second derivatives of the infrared spectra (see Figure S7a,b). For both the untreated and hydrated samples, we resolved two minima around 1626 and 1656 cm^–1^ corresponding to β-sheet and helical secondary structures, in good agreement with the 2D-IR results. As a further consistency check, we also fitted the linear absorption spectra for the untreated and hydrated samples using Gaussian profiles and fixed their central frequencies at the peak positions found in our 2D-IR measurements. As can be observed in Figure S8a,b, this assignment allows us to nicely reproduce the experimental data, demonstrating that the interpretation of the 2D-IR spectra is consistent with the FTIR spectra. Figure S9 shows the normalized areas extracted from the multipeak fits: given the high correlation between adjacent bands, no quantitative analysis is possible.

In light of our results, we caution researchers using multipeak fitting analysis into making the assumption, based on previous studies, that an increase in the vibrational region between 1620 and 1640 cm^–1^ is mostly determined by the increase of β-sheet content, especially at the early stages of the hydration process.

## Conclusions

In this paper, we show that 2D-IR spectroscopy can be used to disentangle the secondary structures in a complex and unpurified biomaterial such as silk film in a label-free and noninvasive manner. From polarization-difference 2D-IR spectra with the appropriate weighting factors, we obtain 2D-IR spectra where we identify and isolate cross-peak or diagonal peaks. Because helical and β-sheet structures have specific cross-peak patterns, we can assign vibrational bands to specific secondary structures. In the same way, by exploiting the same polarization dependency, we can also remove the cross-peaks that overlap with diagonal peaks, reducing the spectral congestion in 2D-IR and thus enhancing our spectral resolution. This enables us to resolve the presence of an additional vibrational band, which is assigned to the random coil. Thanks to this enhanced resolution, we find that at ambient humidity, the dominant conformation is helical in the film studied here, while β-sheet and random coil structures are present with lower abundance. Upon exposure to high humidity, we find that helical content decreases, while the content of the β-sheet/random coil increases. By comparing the relative magnitudes of the β-sheet cross-peaks, we find that the β-sheet content does not significantly change, implying that in the sample analyzed in this paper, the helical structure mainly converts to the random coil. We thus show that (1) we can disentangle the 2D-IR spectra of unpurified silkworm films, resolving the presence of definite vibrational bands, and, in the case of β-sheet and helical structures, (2) assign the vibrational bands to specific secondary structures and (3) determine their relative change when exposing the silkworm film to humidity without peak fitting.

The molecular properties of the building blocks of hierarchical biomaterials determine the physical properties that are required to fulfill their biological functionalities. Understanding the connection between molecular and macroscopic properties is thus a key determinant to elucidate the success and failure of biomaterials. Here, we showed that 2D-IR can be applied successfully to gain a better structural understanding of the building blocks of unpurified biomaterials. Further application of 2D-IR in combination with other techniques, such as rheology, should enable us to gain a better understanding of the critical relationship between biomechanical and biomolecular properties.

## Supporting Information Available

Simulated parallel and isotropic 2D-IR spectrum; linear IR spectrum; amide II spectral fit; and comparison of 2D-IR signal/IR signal (PDF)

## References

[ref1] Fratzl P.; Weinkamer R. Nature’s hierarchical materials. Prog. Mater. Sci. 2007, 52, 1263–1334. 10.1016/j.pmatsci.2007.06.001.

[ref2] Wegst U. G.; Bai H.; Saiz E.; Tomsia A. P.; Ritchie R. O. Bioinspired structural materials. Nat. Mater. 2014, 14, 23–36. 10.1038/NMAT4089. 25344782

[ref3] Holland C.; Numata K.; Rnjak-Kovacina J.; Seib F. P. The Biomedical Use of Silk: Past, Present, Future. Adv. Healthcare Mater. 2019, 8, 1800465 10.1002/adhm.201800465. 30238637

[ref4] Du N.; Xiang Y. L.; Narayanan J.; Li L.; Lim M. L. M.; Li D. Design of Superior Spider Silk: From Nanostructure to Mechanical Properties. Biophys. J. 2006, 91, 4528–4535. 10.1529/biophysj.106.089144. 16950851 PMC1779941

[ref5] Nova A.; Keten S.; Pugno N. M.; Redaelli A.; Buehler M. J. Molecular and Nanostructural Mechanisms of Deformation, Strength and Toughness of Spider Silk Fibrils. Nano Lett. 2010, 10, 2626–2634. 10.1021/nl101341w. 20518518

[ref6] Takahashi Y.; Gehoh M.; Yuzuriha K. Structure refinement and diffuse streak scattering of silk (*Bombyx mori*). Int. J. Biol. Macromol. 1999, 24, 127–138. 10.1016/S0141-8130(98)00080-4. 10342756

[ref7] Lefèvre T.; Rousseau M. E.; Pézolet M. Protein Secondary Structure and Orientation in Silk as Revealed by Raman Spectromicroscopy. Biophys. J. 2007, 92, 2885 10.1529/biophysj.106.100339. 17277183 PMC1831708

[ref8] Laity P. R.; Holland C. Seeking Solvation: Exploring the Role of Protein Hydration in Silk Gelation. Molecules 2022, 27, 551 10.3390/molecules27020551. 35056868 PMC8781151

[ref9] Asakura T.; Suzuki H.; Watanabe Y. Conformational characterization of silk fibroin in intact *Bombyx mori* and Pilosamia cynthia ricini silkworms by carbon-13 NMR spectroscopy. Macromolecules 1983, 16, 1024–1026. 10.1021/ma00240a043.

[ref10] Asakura T.; Watanabe Y.; Uchida A.; Minagawa H. NMR of silk fibroin. Carbon-13 NMR study of the chain dynamics and solution structure of *Bombyx mori* silk fibroin. Macromolecules 1984, 17, 1075–1081. 10.1021/ma00135a017.

[ref11] Iizuka E.; Yang J. T. The disordered and β-conformations of silk fibroin in solution. Biochemistry 1968, 7, 2218–2228. 10.1021/bi00846a026. 5660045

[ref12] Asakura T.; Okushita K.; Williamson M. P. Analysis of the structure of *Bombyx mori* silk fibroin by NMR. Macromolecules 2015, 48, 2345–2357. 10.1021/acs.macromol.5b00160.

[ref13] Barth A. What Vibrations Tell Us About Proteins. Q. Rev. Biophys. 2002, 35, 369–430. 10.1017/S0033583502003815. 12621861

[ref14] Hahn S.; Kwak K.; Cho M. Two-dimensional vibrational spectroscopy. IV. Relationship between through-space vibrational coupling and intermolecular distance. J. Chem. Phys. 2000, 112, 4553–4556. 10.1063/1.481014.

[ref15] Cha S.; Ham S.; Cho M. Amide I vibrational modes in glycine dipeptide analog: Ab initio calculation studies. J. Chem. Phys. 2002, 117, 740–750. 10.1063/1.1483257.

[ref16] Cheatum C. M.; Tokmakoff A.; Knoester J. Signatures of beta-sheet secondary structures in linear and two-dimensional infrared spectroscopy. J. Chem. Phys. 2004, 120, 8201–8215. 10.1063/1.1689637. 15267740

[ref17] Cunha A. V.; Bondarenko A. S.; Jansen T. L. C. Infrared spectroscopy of proteins. Biochim. Biophys. Acta, Bioenerg. 2007, 1073–1101, 1767 10.1016/j.bbabio.2007.06.004. 17692815

[ref18] Higgs P. W. The vibrational spectra of helical molecules: infra-red and Raman selection rules, intensities and approximate frequencies. Proc. R. Soc. London, Ser. A 1953, 133, 472–485. 10.1098/rspa.1953.0200.

[ref19] Baronio C. M.; Baldassarre M.; Barth A. Insight into the internal structure of amyloid-β oligomers by isotope-edited Fourier transform infrared spectroscopy. Phys. Chem. Chem. Phys. 2019, 21, 8587–8597. 10.1039/C9CP00717B. 30964131

[ref20] Paquet-Mercier F.; Lefévre T.; Auger M.; Pézolet M. Evidence by infrared spectroscopy of the presence of two types of β-sheets in major ampullate spider silk and silkworm silk. Soft Matter 2013, 9, 208–215. 10.1039/C2SM26657A.

[ref21] Miyazawa T.; Shimanouchi T.; Mizushima S.-I. Normal vibrations of *N*-methylacetamide. J. Chem. Phys. 1958, 29, 611–616. 10.1063/1.1744547.

[ref22] Hu X.; Kaplan D.; Cebe P. Determining Beta-Sheet Crystallinity in Fibrous Proteins by Thermal Analysis and Infrared Spectroscopy. Macromolecules 2006, 39, 6161–6170. 10.1021/ma0610109.

[ref23] Cho M. Coherent Two-dimensional optical spectroscopy. Chem. Rev. 2008, 108, 1331–1418. 10.1021/cr078377b. 18363410

[ref24] Hunt N. T. 2D-IR spectroscopy: ultrafast insights into biomolecule structure and function. Chem. Soc. Rev. 2009, 38, 1837–1848. 10.1039/b819181f. 19551165

[ref25] Hamm P.; Zanni M. Concepts and Methods of 2D Infrared Spectroscopy; Cambridge University Press: Cambridge, 2011.

[ref26] Minnes L.; Shaw D. J.; Cossins B. P.; Donaldson P. M.; Greetham G. M.; Towrie M.; Parker A. W.; Baker M. J.; Henry A. J.; Taylor R. J.; Hunt N. T. Quantifying Secondary Structure Changes in Calmodulin Using 2D-IR Spectroscopy. Anal. Chem. 2017, 89, 10898–10906. 10.1021/acs.analchem.7b02610. 28921967

[ref27] Fritzsch R.; Hume S.; Minnes L.; Baker M. J.; Burley G. A.; Hunt N. T. Two-dimensional infrared spectroscopy: an emerging analytical tool?. Analyst 2020, 145, 2014–2024. 10.1039/C9AN02035G. 32051976

[ref28] Donaldson P. M. Photon echoes and two dimensional spectra of the amide I band of proteins measured by femtosecond IR and Raman spectroscopy. Chem. Sci 2020, 11, 8862–8874. 10.1039/D0SC02978E. 34123140 PMC8163424

[ref29] Hill R. E.; Hunt N. T.; Hirst J. D. Studying Biomacromolecules with Two-Dimensional Infrared Spectroscopy. Adv. Protein Chem. Struct. Biol. 2013, 93, 1–36. 10.1016/B978-0-12-416596-0.00001-4. 24018321

[ref30] Ernst R. R.; Bodenhausen G.; Wokaun A. Principles of Nuclear Magnetic Resonance in One and Two Dimensions; Clarendon Press: Oxford, 1987.

[ref31] Ashihara S.; Huse N.; Espagne A.; Nibbering E. T.; Elsaesser T. Vibrational couplings and ultrafast relaxation of the O–H bending mode in liquid H_2_O. Chem. Phys. Lett. 2006, 424, 66–70. 10.1016/j.cplett.2006.04.051.

[ref32] Huerta-Viga A.; Shaw D. J.; Woutersen S. pH Dependence of the Conformation of Small Peptides Investigated with Two-Dimensional Vibrational Spectroscopy. J. Phys. Chem. B 2010, 114, 15212–15220. 10.1021/jp105133r. 20977228

[ref33] Roeters S. J.; van Dijk C.; Torres-Knoop A.; Backus E. H.; Campen R. K.; Bonn M.; Woutersen S. Determining in situ protein conformation and orientation from the amide-I sum-frequency generation spectrum: theory and experiment. J. Phys. Chem. A 2013, 117, 6311–6322. 10.1021/jp401159r. 23566310

[ref34] Krimm S.; Bandekar J. Vibrational spectroscopy and conformation of peptides, polypeptides, and proteins. Adv. Protein Chem. 1986, 38, 181–364. 10.1016/S0065-3233(08)60528-8. 3541539

[ref35] Strazdaite S.; Roeters S. J.; Sakalauskas A.; Sneideris T.; Kirschner J.; Pedersen K. B.; Schiøtt B.; Jensen F.; Weidner T.; Smirnovas V.; Niaura G. Interaction of Amyloid--(1–42) Peptide and Its Aggregates with Lipid/Water Interfaces Probed by Vibrational Sum-Frequency Generation Spectroscopy. J. Phys. Chem. B 2021, 125, 11208–11218. 10.1021/acs.jpcb.1c04882. 34597059

[ref36] Robustelli P.; Piana S.; Shaw D. E. Developing a molecular dynamics force field for both folded and disordered protein states. Proc. Natl. Acad. Sci. U.S.A 2018, 115, E4758–E4766. 10.1073/pnas.1800690115. 29735687 PMC6003505

[ref37] Askarieh G.; Hedhammar M.; Nordling K.; Saenz A.; Casals C.; Rising A.; Johansson J.; Knight S. D. Self-assembly of spider silk proteins is controlled by a pH-sensitive relay. Nature 2010, 465, 236–238. 10.1038/nature08962. 20463740

[ref38] Pettersen E. F.; Goddard T. D.; Huang C. C.; Couch G. S.; Greenblatt D. M.; Meng E. C.; Ferrin T. E. UCSF Chimera—a visualization system for exploratory research and analysis. J. Comput. Chem. 2004, 25, 1605–1612. 10.1002/jcc.20084. 15264254

[ref39] Panman M. R.; van Dijk C. N.; Meuzelaar H.; Woutersen S. Communication: Nanosecond folding dynamics of an alpha helix: Time-dependent 2D-IR cross peaks observed using polarization-sensitive dispersed pump-probe spectroscopy. J. Chem. Phys. 2015, 142, 01B401_1 10.1063/1.4906456. 25637962

[ref40] Woutersen S.; Hamm P. Structure determination of trialanine in water using polarization sensitive two-dimensional vibrational spectroscopy. J. Phys. Chem. B 2000, 104, 11316–11320. 10.1021/jp001546a.

[ref41] Zanni M. T.; Ge N. H.; Kim Y. S.; Hochstrasser R. M. 2D-IR can be designed to eliminate the diagonal peaks and expose only the crosspeaks needed for structure determination. Proc. Natl. Acad. Sci. U.S.A. 2001, 98, 11265–11270. 10.1073/pnas.201412998. 11562493 PMC58718

[ref42] Woutersen S.; Hamm P. Time-resolved two-dimensional vibrational spectroscopy of a short α-helix in water. J. Chem. Phys. 2001, 1151, 2727–7733. 10.1063/1.1336807.

[ref43] Panman M. R.; van Dijk C. N.; Meuzelaar H.; Woutersen S. Communication: Nanosecond folding dynamics of an alpha helix: Time-dependent 2D-IR cross peaks observed using polarization-sensitive dispersed pump-probe spectroscopy. J. Chem. Phys. 2015, 142, 041103 10.1063/1.4906456. 25637962

[ref44] Asakura T. Structure of Silk I (*Bombyx mori* Silk Fibroin before Spinning) -Type II ß-Turn, Not -Helix-. Molecules 2021, 26, 3706 10.3390/molecules26123706. 34204550 PMC8234240

[ref45] Barth A. Infrared spectroscopy of proteins. Biochim. Biophys. Acta, Bioenerg. 2007, 1767, 1073–1101. 10.1016/j.bbabio.2007.06.004. 17692815

[ref46] Zanni M. T.; Asplund M. C.; Hochstrasser R. M. Two-dimensional heterodyned and stimulated infrared photon echoes of N-methylacetamide-D. J. Chem. Phys. 2001, 114, 4579–4590. 10.1063/1.1346647.

[ref47] Yazawa K.; Ishida K.; Masunaga H.; Hikima T.; Numata K. Influence of Water Content on the β-Sheet Formation, Thermal Stability, Water Removal, and Mechanical Properties of Silk Materials. Biomacromolecules 2016, 17, 1057–1066. 10.1021/acs.biomac.5b01685. 26835719

