# Supplementary material for: In Situ Identification of Secondary Structures in Unpurified Bombyx mori Silk Fibrils Using Polarized Two-Dimensional Infrared Spectroscopy
Source: Biomacromolecules. 2022 Nov 28;23(12):5340–9. doi: 10.1021/acs.biomac.2c01156 (PMC9748943; doi:10.1021/acs.biomac.2c01156)
Supplement: Supplementary file 1 — bm2c01156_si_001.pdf [file bm2c01156_si_001.pdf]

Supplementary for:
In situ identification of secondary structures in unpurified silkworm films using polarized advanced infrared spectroscopy

Giulia Giubertoni,[*,†,‡,‖] Federico Caporaletti,[*,‡,†,‖] Steven J. Roeters,[¶,†] Adam S. Chatterley,[¶] Tobias Weidner,[¶] Peter Laity,[§] Chris Holland,[§] and Sander Woutersen[†]

† *Van 't Hoff Institute for Molecular Sciences, University of Amsterdam, Science Park 904, 1098XH Amsterdam, The Netherlands*

‡ *Van der Waals-Zeeman Institute, Institute of Physics, University of Amsterdam, 1098XH Amsterdam, The Netherlands*

¶ *Department of Chemistry, Aarhus University, Aarhus C, Denmark*

§ *Department of Materials Science and Engineering, University of Sheffield, Sir Robert Hadfield Building, Mappin St., Sheffield S1 3JD, UK*

‖ *These authors contributed equally to the work*

E-mail: g.giubertoni@uva.nl; f.caporalettii@uva.nl

FTIR spectra of the hydrated silkworm films

Fig. S1 shows the extended IR-spectra of the untreated and hydrated silkworm films. The silkworm films have been exposed to an environment saturated with D_2O instead of H_2O to suppress the spectral contributions of the H-O-H bending mode of water, which would have masked the amide region of the spectrum.To take in consideration the fact that we probed different spots of the sample with different thicknesses before and after the hydration, the spectra of the untreated and hydrated samples were normalized to the area of the C-H region which absorbs around 2936 cm-1. After the treatment a broad vibrational band, corresponding to the O-D stretching of water, appears around 2500 cm^{-1}, while the band corresponding to the N-H stretching mode decreases following deuteration. The amide groups

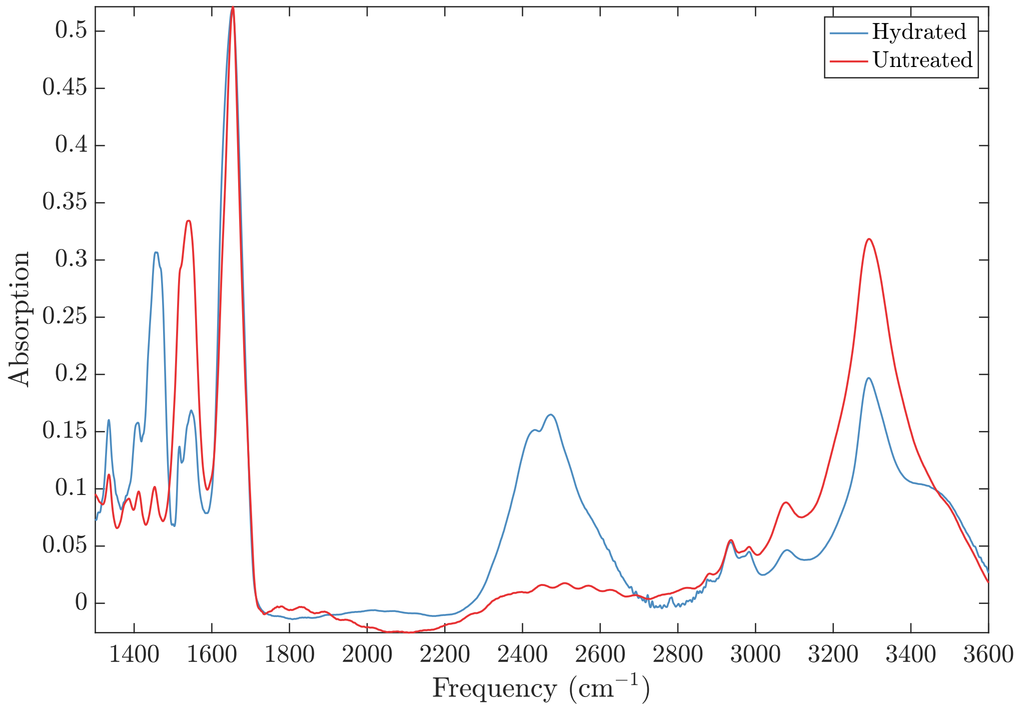

Figure S1: Normalized linear infrared spectra hydrated and untreated silk film produced by Bombyx Caterpillars. The exposure to a environment saturated of D_2O leads to the appearance of the O-D stretch vibrational band around 2500 cm^{-1}.

that are able to isotopically exhange H with D during the hydration process are those that

are water accessible. Upon H/D exchange, the amide II shifts to lower frequency, absorbing around 1450 cm^{-1} and we refer to it as amide II'. The area of the vibrational band of the amide II' hence reflects the amount of groups in the film that can interact with water. Fig. S2 shows the amide II' and amide II vibrational bands for Sample A discussed in the main text: from the calculated areas we can estimate that $\simeq$ 70% of the NH groups are H/D exchanged to ND.

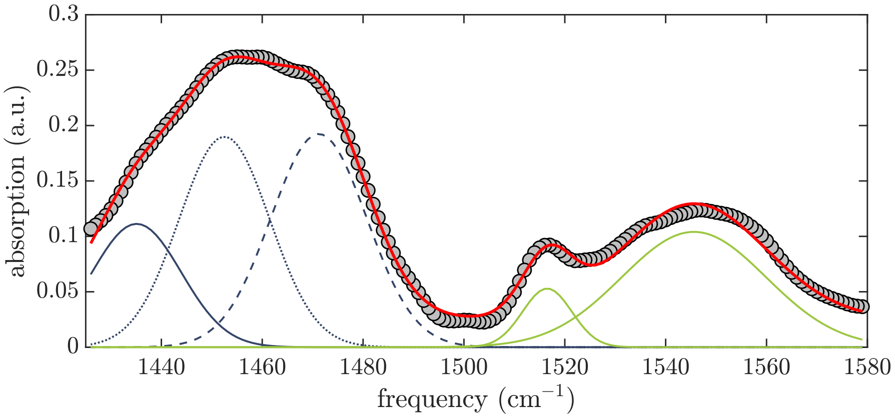

Figure S2: Fit of the linear spectrum of hydrated silk film in the amide II region. To estimate the amount of amide groups that isotopically exchange (i.e. that are water accessible), we extract the areas of vibrational bands of the amide II', and amide II. To obtain a quantitatively good fit, we use three Gaussian-shaped peaks to describe the amide II absorption bands at 1450 cm^{-1}, and 2 Gaussian-shaped peaks to described the amide II at 1550 cm^{-1}.

Anisotropy

To obtain information over the molecular orientation, we calculate the anisotropy, defined as $R = \frac{\Delta\alpha_{\text{par}} - \Delta\alpha_{\text{per}}}{\Delta\alpha_{\text{par}} + 2\Delta\alpha_{\text{per}}}$, where $\Delta\alpha_{\text{par}}$ and $\Delta\alpha_{\text{per}}$ are the transient absorption changes measured in parallel and in perpendicular polarization configuration, respectively. In case of the diagonal peaks, the ratio between parallel and perpendicular signal is expected to be 3, leading to an anisotropy of 0.4. In Fig.S3, we report the anisotropy as a function of probe frequency obtained by centering the excitation pulse at the beta-sheet $A_{\perp}$ vibrational mode, which absorbs at 1623 cm^{-1}. We observe that at the probe frequency of the same mode, where the bleach of the diagonal peak is found in the 2DIR spectrum, the anisotropy value is perfectly at 0.4, indicating that the scaling factor between parallel and perpendicular is 3 in our experiments. We also observe that at 1710 cm^{-1}, where the β-sheet $A_{\parallel}$ vibrational mode absorbs, the anisotropy changes to negative values, approaching -0.2. In this case, the anisotropy represents the relative orientation of the $A_{\perp}$ transition dipole moment with respect to the $A_{\parallel}$ dipole moment. We can calculate the relative angle $\theta = \arccos\sqrt{\frac{5R_0+1}{3}}$, obtaining an angle of around 90°, which is expected since $A_{\perp}$ and $A_{\parallel}$ have transition dipole moments that lie perpendicular to each other.

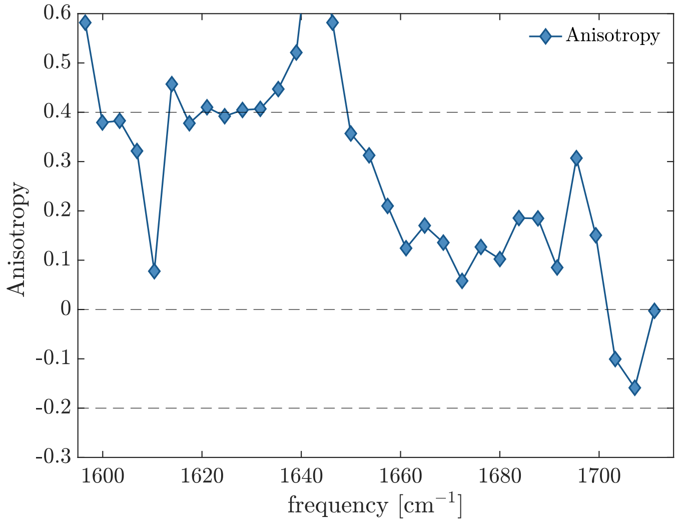

Figure S3: Anisotropy values as a function of probe frequency upon excitation of th e β-sheet $A_{\perp}$ vibrational mode. At certain frequencies, such as 1645 cm^{-1}, the anisotropy values are out of scale because these are the frequencies of the nodal line between the negative and positive peaks, where the parallel and perpendicular signals are ~ 0.

Absence of β-sheet secondary structures in untreated films

To confirm that the helical structure is the most abundant structure in untreated silk films, we measured another film that was prepared with the same protocol using a native silk feedstock liquid extracted from a different gland. Fig. S4 shows the "diagonal-free" spectrum this silk film before being hydrated. We here observe only the off-diagonal signatures of the helical structure, confirming that in silkworm films proteins adopt mostly helical conformation at ambient conditions.

Anti-diagonal slices

In Fig.S5 we report the diagonal free 2DIR spectra and the anti-diagonal slices from where we obtain the 2DIR signals reported in the main text.

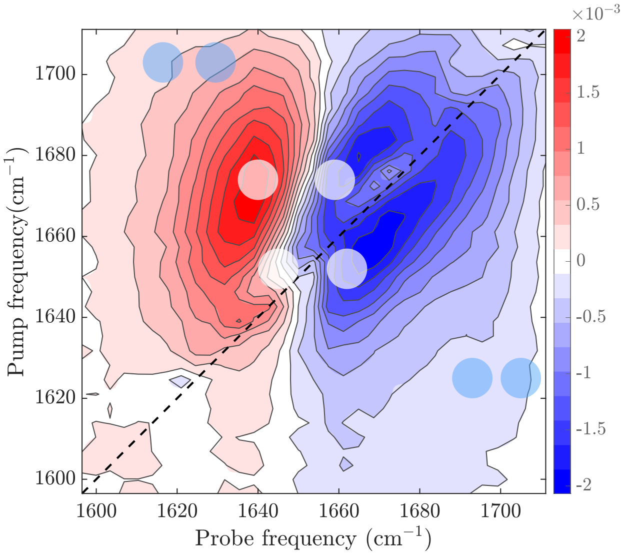

Figure S4: Subtracted 2D-IR spectrum of an untreated silk film, coming from a different batch than the sample discussed in the main text. The spectrum displays the cross-peaks associated to helical secondary structures (see white circles), while the cross-peaks signatures expected for β-sheet, which would be expected at the frequencies highlighted by the blue circles, are absent.

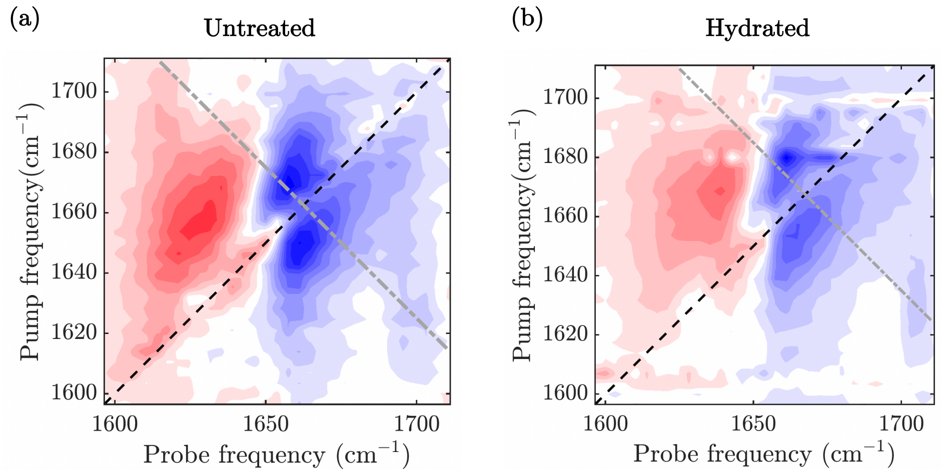

Figure S5: Diagonal free 2D-IR spectra of the untreated (a) and hydrated (b) films discussed in the main text. The main diagonal (black dashed line) and anti-diagonal slice (gray dashed-dotted line), used in Fig. 5 are highlighted.

Effect of exposure time to high humidity on β-sheet content

The experimental data reported in this section refers to a sample from the same batch as the one shown in the main text and were measured at the University of Aarhus using

a 10 kHz commercial time-domain 2DIR spectrometer (PhaseTech 2DQuickIR) described previously.[1,2] Briefly, femtosecond mid-IR pulses were split into pump and probe pulses. The pump pulses were split into time- and phase-controlled pulse pairs using an acousto-optic pulse shaper, and then focused into the sample 500 fs before the probe pulse, which was dispersed onto an MCT detector (PhaseTech JackHammer). The delay between the pump pulse varied in 33 fs steps from 0 to 3 ps, and the measurements were performed in a 1300 cm-1 rotating frame. In order to reduce interference from scattered light, a 4-frame phase cycling scheme was used, and reference spectra recorded at -10 ps were subtracted. The sample here analyzed is taken from the same silkworm film on which the experiments

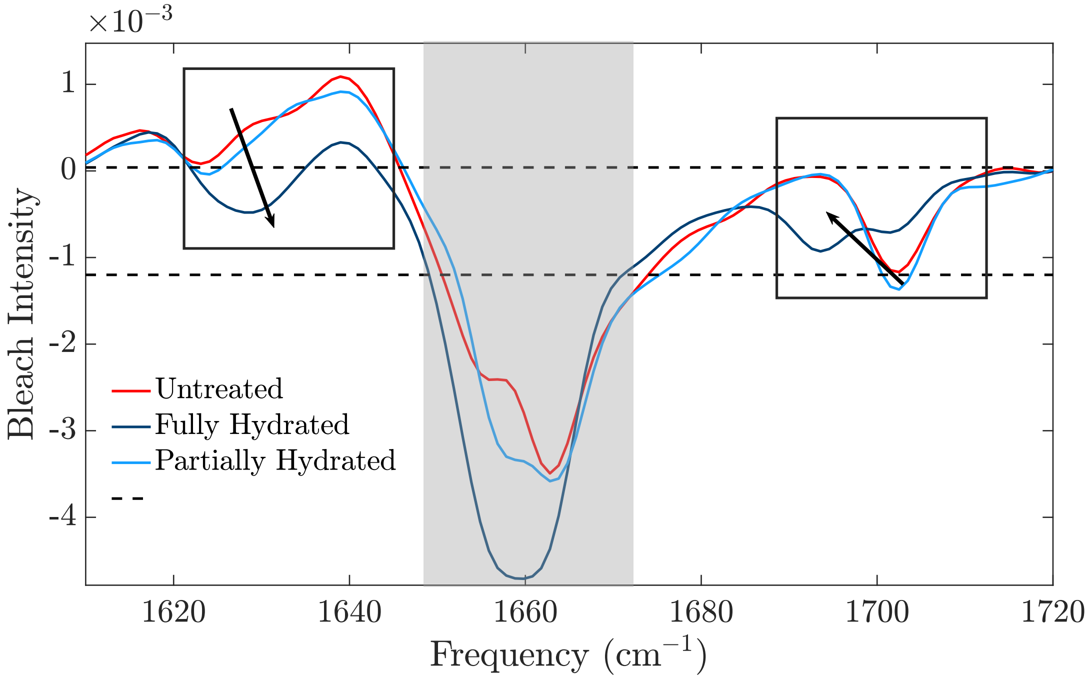

Figure S6: Anti-diagonal slice of the diagonal free spectrum measured from a silkworm film from the same batch as in the main text at different level of hydration: untreated (red solid line), hydrated for 2 hours (cyan solid line) and for 36 hours (blue solid line). The black boxes highlight the changes occurring in the cross-peaks associated to β-sheet secondary structures. The gray rectangle shadows the helical region, close to the main diagonal, that is affected by scattering in the shown measurement.

reported in the main text were performed. After exposing the film to saturated D_2O (85% RH) no major change is observed in magnitude of the cross-peaks between the β-sheet modes

at 1630 cm^{-1}, and at 1700 cm^{-1}, indicating that the β-sheet content is constant. This confirms and well-reproduces the result presented in the main text. We further expose this sample to high humidity for additional 34 hours. Upon this, the cross-peaks between the β-sheet modes change: the magnitude of the cross-peak at 1620 cm^{-1} increases drastically, while the cross-peak at 1700 cm^{-1} spits in two distinct subbands. This last effect is due to the overlap of different cross-peaks, likely associated to the hydrated and not-hydrated β-sheet. Because of the overlap, the relative intensities of the cross-peaks cancel partially off, and thus we do not consider it in our analysis. However, it is clearly visible that the magnitude of the cross-peak at 1620 cm^{-1} increases drastically, indicating that β-sheet structures are being formed after long exposure time (see blue curve in Fig. S6).

Analysis of linear IR spectra

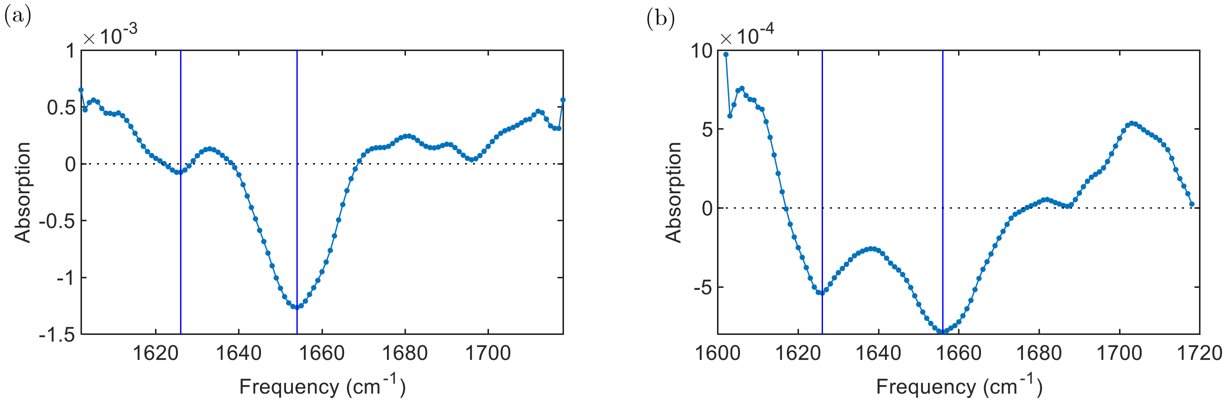

Figure S7: Second derivative of the linear infrared spectra of the untreated (a) and hydrated (b) silk film. The blue vertical lines are a guide for the eye to identify the main minima.

In this section, we reports additional analysis of the linear infrared spectra shown in Fig. 1 of the manuscript as further consistency check of the results obtained from the 2D-IR measurements .

Second derivative

Fig. S7-(a,b) shows the second derivative of the spectra of the untreated and hydrated silk films, respectively. It is possible to observe two significant minima around 1626 cm^{-1} and 1656 cm^{-1}, which are the values provided by the analysis of the 2D-IR spectra.

Multi-peak fitting

The linear absorption spectra (Fig. 1 of the main text), were fitted using 5 Gaussian-shaped peaks absorbing at 1627, 1640, 1658, 1680 and at 1550 cm^{-1}. The fits are performed by leaving the width of the peaks as free parameters, while we set boundaries on the center frequencies based on the 2DIR data. As shown in Fig. S8, the fits well reproduce the linear infrared data. The relative percentages of each secondary structure, extracted from the multi-peak fitting are plotted in Fig.S9: the uncertainties, arising from the high cross-correlation between the different bands, do not allow a reliable estimate.

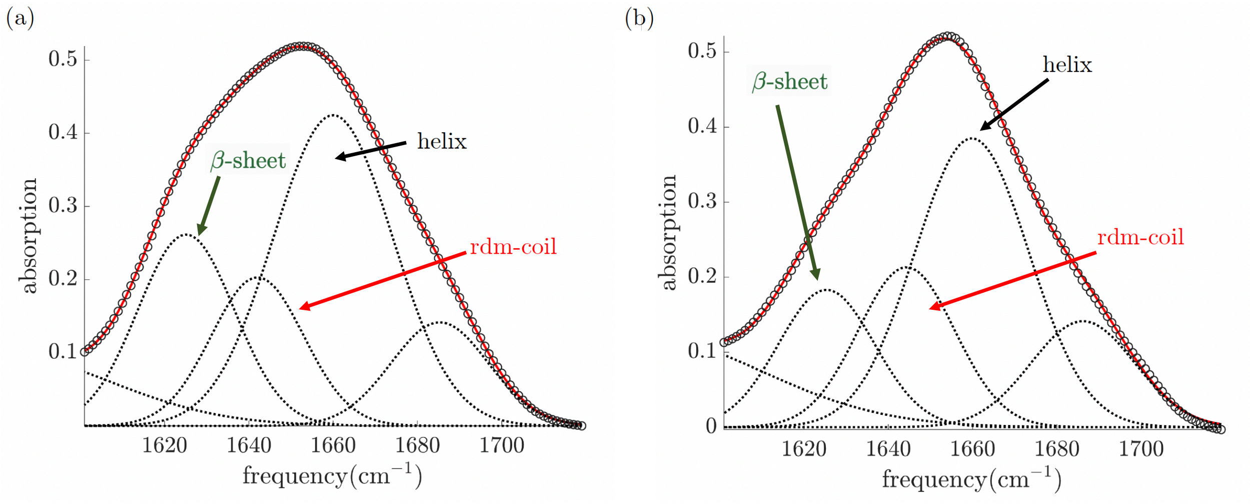

Figure S8: Multi-peak fitting of the congested amide-I band of the linear IR-spectra of the hydrated (a) and untreated spectra (b) shown in Fig. 2 of the main text. Five Gaussians, with central frequencies fixed from the 2D-IR measurements are used to model the absorption band. The main bands (β-sheet, helix and random coild) are directly indicated in the figure, while the low frequency tail and the high frequency peak are contributions from the amide II band and β-turn, respectively.

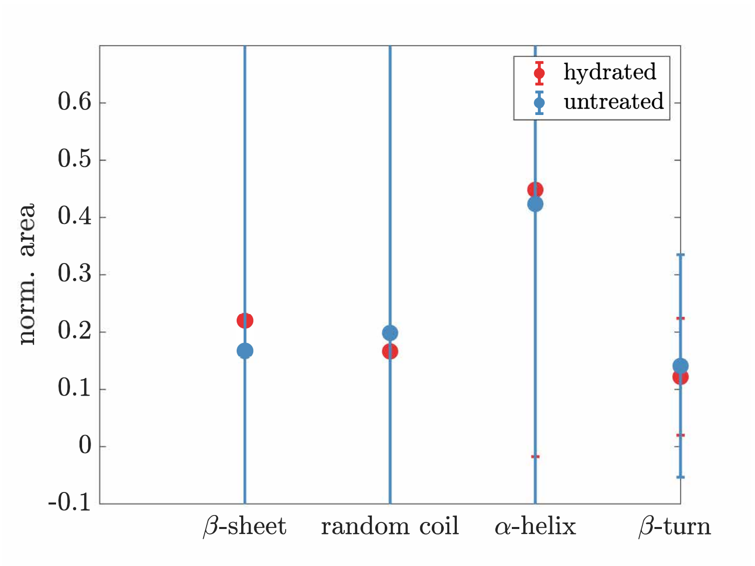

Figure S9: Relative percentage of the different spectral contributions to the IR amide band of the untreated (red circles with errorbars) and hydrated (blue circles with errorbars) films, extracted from multi-peak fitting.

Measurement reproducibility

In this section, we report the full analysis of the 2D-IR data collected on another silk worm film, from the same batch of the one analyzed in the main text. These additional data were collected at the University of Aarhus (see also Fig. S6) using 0.5 ps delay between the pump and probe pulse. The sample was hydrated using the same procedure reported in the main text. Fig. S10 reports the perpendicular (a,b) , diagonal-free (c,d) and cross-peak free (d,e) spectra of the untreated and partially hydrated film, respectively. It is possible to observe that the main results presented in the manuscript are nicely reproduced. In particular, it is possible to observe in Fig.S11 that upon partial hydration the β-sheet content does not increase, as also indicated by the lack of major modifications in the anti-diagonal slices (Fig. S6). On the contrary there is an strong increase in the diagonal slice (see Fig. S11) around 1650 cm^{-1}, compatible with random-coil secondary structures. This feature is more visible in this second dataset because the data were collected at a shorter time (0.5 ps), where the contribution of the random coil to the bleach is expected to be stronger because the random coil has a shorter vibrational lifetime than the β-sheet.[3]

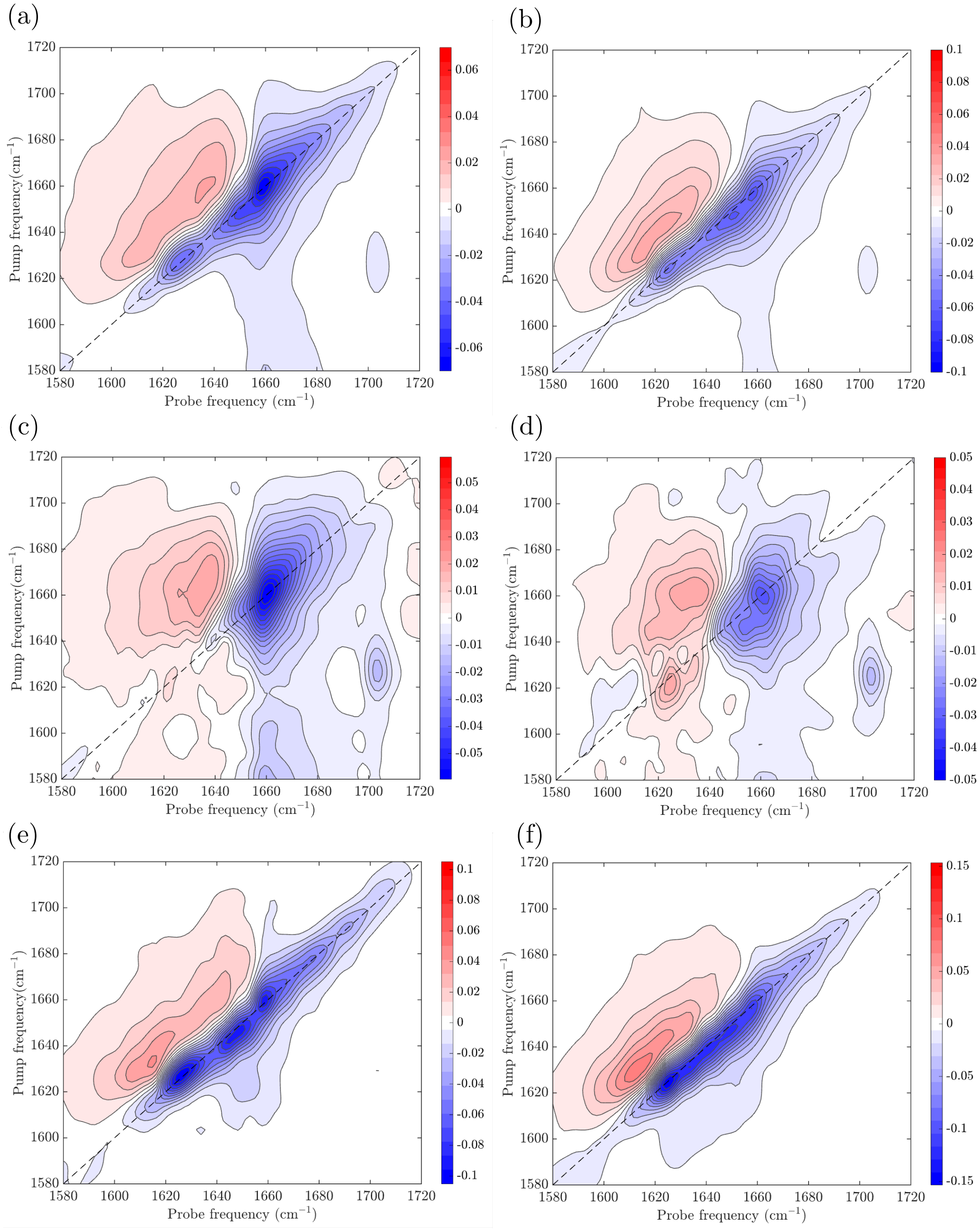

Figure S10: Perpendicular (a,b), diagonal-free (c,d) and cross-peak free spectra (e,f) measured with a pump-probe delay at 0.5 ps for an untreated and hydrated silkworm film. The data were collected at the University of Aarhus using a pump-probe delay of 0.5 ps.

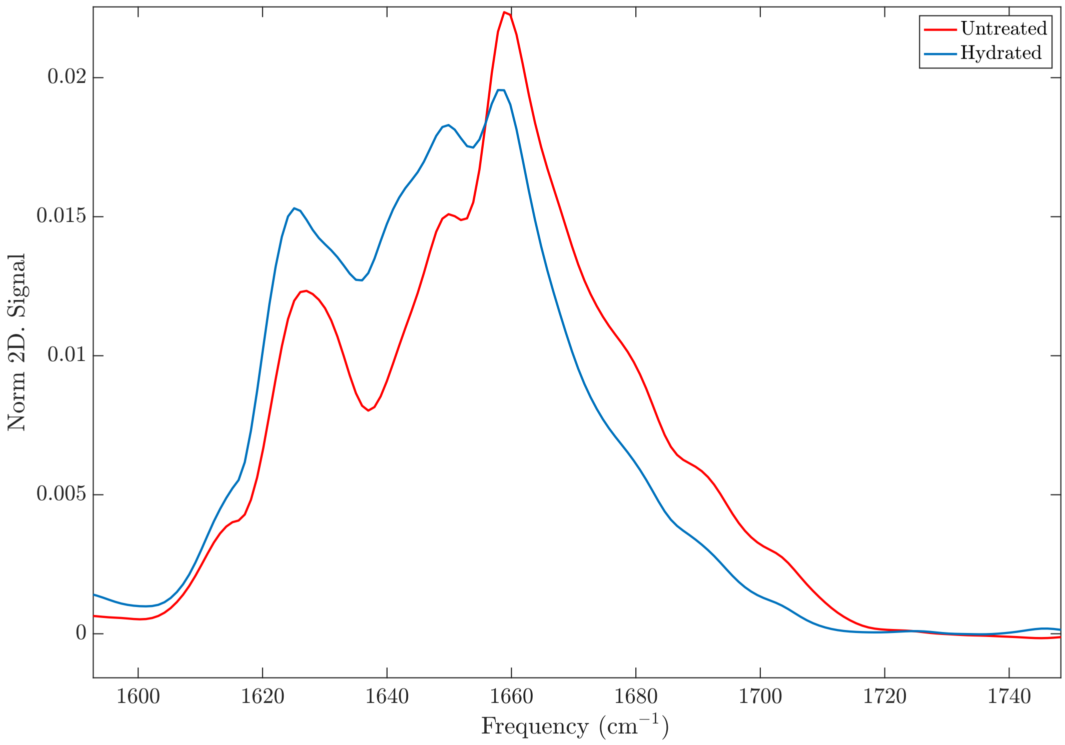

Figure S11: Untreated (red solid line) and treated (blue solid line) diagonal slices of the cross-peak free spectra shown in Fig. S10-(e,f).

References

(1) Shim, S.-H.; Zanni, M. T. How to turn your pump–probe instrument into a multidimensional spectrometer: 2D IR and Vis spectroscopies via pulse shaping. *Phys. Chem. Chem. Phys.* **2009**, *11*, 748–761.

(2) Madzharova, F.; Bregnhøj, M.; Chatterley, A. S.; Løvschall, K. B.; Drace, T.; Andersen Dreyer, L. S.; Boesen, T.; Weidner, T. Electrostatics Trigger Interfacial Self-Assembly of Bacterial Ice Nucleators. *Biomacromolecules* **2021**,

(3) Laaser, J. E.; Skoff, D. R.; Ho, J.-J.; Joo, Y.; Serrano, A. L.; Steinkruger, J. D.; Gopalan, P.; Gellman, S. H.; Zanni, M. T. Two-dimensional sum-frequency generation reveals structure and dynamics of a surface-bound peptide. *J. Am. Chem. Soc.* **2014**, *136*, 956–962.
